# Effectiveness of Immersive and Non-Immersive Virtual Reality Interventions on Cognitive Function in People with Multiple Sclerosis: A Systematic Review

**DOI:** 10.3390/jcm15124534

**Published:** 2026-06-11

**Authors:** Roberto López-Andaur, Edgar Vásquez-Carrasco, Luisa Guerra-Labbé, Jordan Hernandez-Martinez, Pablo Valdés-Badilla, Cristian Sandoval-Vásquez, Eduardo Carmine-Peña, Constanza Lorca, Ana Belén Calvo-Vera

**Affiliations:** 1 Exercise and Rehabilitation Sciences Institute, School of Occupational Therapy, Faculty of Rehabilitation Sciences, Universidad Andres Bello, Santiago de Chile 7591538, Chile; r.lopezandaur@uandresbello.edu; 2 School of Occupational Therapy, Faculty of Psychology, Universidad de Talca, Talca 3465548, Chile; 3 Centro de Investigación en Ciencias Cognitivas, Faculty of Psychology, Universidad de Talca, Talca 3465548, Chile; 4 VITALIS Longevity Center, Universidad de Talca, Talca 3465548, Chile; 5 Carrera de Terapia Ocupacional, Facultad de Ciencias de la Salud, Universidad Autónoma de Chile, Providencia 7500000, Chile; luisa.guerra@uautonoma.cl; 6 Department of Physical Activity Sciences, Universidad de Los Lagos, Osorno 5290000, Chile; jordan.hernandez@ulagos.cl; 7 Department of Education, Faculty of Humanities, Universidad de la Serena, La Serena 1700000, Chile; 8 Department of Physical Activity Sciences, Faculty of Education Sciences, Universidad Católica del Maule, Talca 3530000, Chile; valdesbadilla@gmail.com; 9 Sports Coach Career, Faculty of Life Sciences, Universidad de Viña del Mar, Viña del Mar 2520000, Chile; 10 Escuela de Tecnología Médica, Facultad de Salud, Universidad Santo Tomás, Los Carreras 753, Osorno 5310431, Chile; cristian.sandoval@ufrontera.cl; 11 Departamento de Medicina Interna, Facultad de Medicina, Universidad de La Frontera, Temuco 4811230, Chile; 12 Carrera de Medicina, Facultad de Medicina, Universidad de La Frontera, Temuco 4811230, Chile; e.carmine01@ufromail.cl; 13 Departamento de Ciencias Básicas, Facultad de Medicina, Universidad de La Frontera, Temuco 4811230, Chile; 14 Departamento de Enfermería y Fisioterapia, Facultad de Enfermería y Fisioterapia, Universidad de Salamanca, 37008 Salamanca, Spain; 15 NeuroUsal Team, 37007 Salamanca, Spain; 16 Salamanca Biomedical Research Institute (IBSAL), 37007 Salamanca, Spain

**Keywords:** multiple sclerosis, virtual reality, rehabilitation cognitive, technology

## Abstract

**Background/Objectives:** Multiple sclerosis (MS) is a chronic neurological disorder affecting more than 2.8 million individuals worldwide and is commonly associated with cognitive deficits that compromise independence and quality of life. In recent years, virtual reality (VR) has emerged as an innovative rehabilitation strategy, offering immersive and engaging environments that promote neuroplasticity and enhance patient motivation. To evaluate the effectiveness of immersive and non-immersive VR-based interventions in improving cognitive performance among adults diagnosed with MS. **Methods:** A systematic review was conducted following the Cochrane Handbook for Systematic Reviews of Interventions and PRISMA 2020 guidelines (PROSPERO registration: CRD420251103762). Comprehensive searches were carried out across seven international databases up to October 2025, including only randomized controlled trials assessing cognitive outcomes after VR-based rehabilitation programs. **Results:** From 1948 records screened, 13 studies comprising 649 participants met the inclusion criteria. Intervention durations ranged between 6 and 17 weeks, with sessions lasting 30–60 min. The interventions involved treadmill training with VR, exergaming, and cognitive stimulation protocols. Most studies demonstrated significant improvements in processing speed, visuospatial and verbal memory, and executive functioning (*p* < 0.05). Adherence rates were above 80%, and no serious adverse events were reported. **Conclusions:** VR-based rehabilitation appears to be a safe, feasible, and effective approach for enhancing cognitive abilities in individuals with MS, particularly in processing speed and visuospatial memory. Nonetheless, the heterogeneity of methodologies underscores the need for standardized intervention frameworks and large-scale multicenter randomized trials to establish optimal parameters and confirm sustained long-term benefits.

## 1. Introduction

Multiple sclerosis (MS) is one of the leading causes of non-traumatic neurological disability in young adults worldwide, affecting approximately 2.8 million people [1,2]. Women are impacted more frequently, in a near 2:1 female-to-male ratio [3]. Typically diagnosed between the ages of 20 and 40, MS substantially affects participation and quality of life [4]. Cognitive impairment, especially in attention, memory, processing speed, and executive functions affects up to 70% of individuals and is regarded as one of the most disabling manifestations [5]. Fatigue, present in nearly 60% of cases, further limits functional performance [1]. Comorbid conditions may aggravate symptom burden and complicate rehabilitation [6].

Cognitive and functional rehabilitation has therefore become a key element in MS management [7]. Cognitive dysfunction in multiple sclerosis has been recognized as one of the most challenging domains for clinical rehabilitation, as there are currently no pharmacological treatments specifically approved to improve the cognitive functions affected in this population [8,9]. Non-pharmacological approaches are increasingly valued for their therapeutic benefits and low risk profiles [10,11]. Among these, virtual reality (VR) offers structured, motivating, and ecologically valid environments for cognitive training. VR interventions can be delivered through immersive systems (head-mounted displays), semi-immersive systems (large projection or multi-screen setups), or non-immersive systems that rely on external screens with lower immersion but greater accessibility [12,13,14]. These approaches collectively support a person-centered rehabilitation model and highlight the importance of early neuropsychological intervention [15].

Evidence suggests that VR may improve cognitive performance by enhancing engagement, multisensory feedback, and real-world task simulation [16,17]. Prior reviews have reported benefits for global cognition and affective symptoms [18], and VR integration into clinical trials has enabled more sensitive behavioral monitoring than conventional assessments [19]. Technology-based rehabilitation involving motivated exercise and continuous feedback, including exergaming and VR, has also demonstrated benefits for aspects of balance and participation, suggesting that these approaches provide multifactorial benefits that can indirectly influence cognitive function through increased engagement with therapy and repetitive practice [20]. However, existing reviews often combine heterogeneous study designs, lack clear differentiation between immersive and non-immersive VR [14,16,21].

To address these gaps, this review focuses exclusively on randomized controlled trials (RCTs), distinguishes VR modalities, and applies rigorous risk-of-bias evaluation. Therefore, this review aimed to synthesize evidence on the effectiveness of immersive and non-immersive VR-based interventions for improving cognitive function in people with MS.

## 2. Materials and Methods

### 2.1. Protocol and Registration

The present systematic review adhered to Cochrane methodological standards [22] and complied with PRISMA reporting requirements (Table S1, [23]). The protocol was registered in PROSPERO (CRD420251103762).

### 2.2. Eligibility Criteria

This systematic review included peer-reviewed original research articles, specifically RCTs, with no restrictions on language or publication date until to October 2025. Study selection was guided by the PICOS (Population, Intervention, Comparator, Outcomes, Study design) framework, as detailed in Table 1. The following sources were excluded: conference abstracts, books and book chapters, editorials, letters to the editor, registered protocols, reviews (systematic or narrative), case reports, and non-randomized studies.

### 2.3. Information Search Process and Database

Searches were conducted in seven databases (Scopus, Web of Science, MEDLINE/PubMed, EBSCOhost, ProQuest, Embase, and the Cochrane Library). MeSH terms and keywords related to multiple sclerosis, virtual reality (including immersive and non-immersive modalities), and the adult population were used. The strategy combined these concepts using OR and AND operators to encompass clinical variants of MS, different types of VR, and descriptors of adults. To ensure methodological rigor, two independent specialists evaluated the inclusion criteria and the selected studies. Eligibility for expert consultation required: (i) a doctoral degree in health sciences and (ii) a publication record involving peer-reviewed articles on cognitive function across various populations in journals indexed by Journal Citation Reports^®^.

Experts were blinded to the specific search strategy to minimize selection bias. As part of the quality control process, an additional verification was performed on 29 October 2025, to identify potential retractions or published errata concerning the included studies.

### 2.4. Study Selection Process and Data Collection

All retrieved records were imported into Mendeley Reference Manager (version 2.116.1; Elsevier, London, UK), and the selection process was depicted through a PRISMA flow diagram. Two reviewers (R.L.-A. and E.V.-C.) independently performed the searches and sequentially screened the titles, abstracts, and full-text articles after removing duplicates. No inconsistencies were noted during this stage. Subsequently, studies considered potentially eligible were examined in greater detail, with exclusions documented according to the predefined eligibility criteria. To ensure methodological transparency and data integrity, two additional reviewers (J.H.-M. and P.V.-B.) independently verified the entire study selection and data extraction procedures. Any discrepancies arising during the expert consultation process were reviewed and discussed until consensus was reached, and when necessary, a third expert was consulted to resolve disagreements (A.B.C-V.).

### 2.5. Methodological Quality Assessment

Only studies categorized as Level 1a evidence, corresponding to RCTs, were considered eligible for inclusion. Research classified under Levels 1b, 2a, 2b, 3a, 3b, 4, or 5 was excluded. The quality rating of each RCT was subject to downgrading when concerns were identified regarding risk of bias, consistency, accuracy, precision, transparency of findings, or potential publication bias [24].

### 2.6. Data Collection Process

Relevant data from each study included in the systematic review were extracted using a standardized data extraction form, following the recommendations of the Cochrane Handbook [25], and managed with Microsoft Excel (version 2506; Microsoft Corporation, Redmond, WA, USA). Data extraction was performed independently by two reviewers (R.L.-A. and E.V.-C.), who later compared their individual results. The entire data extraction process was jointly supervised (A.B.C.V.). Extracted variables from each study included: title, author/year, country of origin, level of evidence, study design, risk of bias, population and sample size, inclusion criteria, study setting, intervention and control groups, outcome measures, and main findings.

### 2.7. Risk of Bias

The risk of bias in the RCTs included in this systematic review was assessed using the Risk of Bias 2 (RoB 2) tool [19]. Two authors (R.L.-A. and E.V.-C.) independently conducted the assessments, which were subsequently reviewed by two additional authors (J.H.-M. and A.B.C.V.). Any discrepancies in the initial evaluations were addressed by re-examining the original articles, and disagreements were resolved through consensus.

### 2.8. Meta-Analysis Measures

A meta-analysis was not possible due to the marked methodological heterogeneity among the included studies. The main sources of variability included substantial differences in the cognitive assessment instruments used; variations in the duration, frequency, and intensity of VR-based interventions; the use of different VR modalities, immersive and non-immersive and differences in the comparator groups, conventional rehabilitation, telerehabilitation, or no intervention. This heterogeneity prevented the collection of pooled quantitative estimates and justified the adoption of a narrative synthesis.

### 2.9. Certainty of Evidence

The certainty of the evidence for each outcome was assessed using the Grading of Recommendations, Assessment, Development, and Evaluations (GRADE) approach [25]. Since only randomized controlled trials were included, the initial quality of evidence was rated as high. However, the level of certainty was downgraded when concerns arose regarding risk of bias, inconsistency, indirectness, imprecision, or publication bias. Two reviewers (R.L.-A. and E.V.-C.) independently performed the GRADE evaluations, and disagreements were resolved through discussion and consensus with a third reviewer (P.V.-B.).

## 3. Results

### 3.1. Study Selection

**Figure 1:**
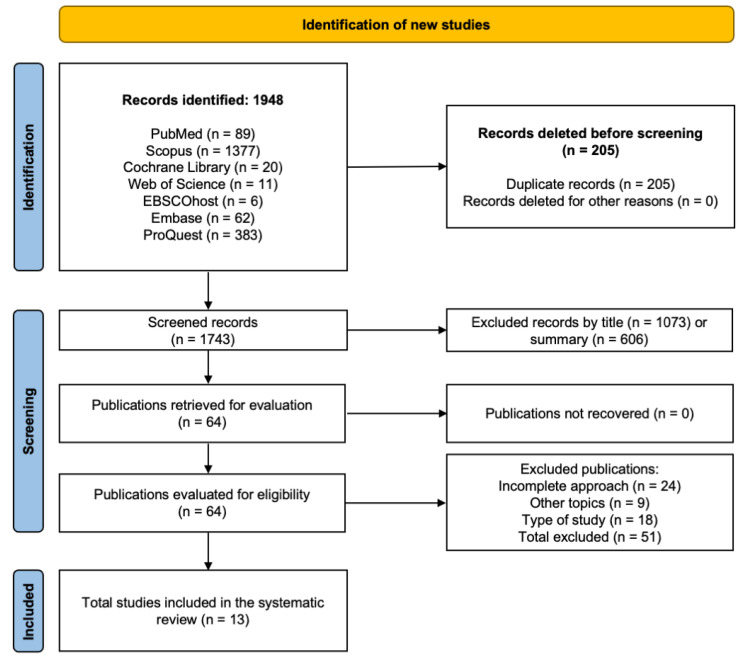
PRISMA flow diagram illustrating the study selection process for the systematic review.

A total of 1948 studies were identified in all databases (PubMed, Scopus, ProQuest, Cochrane Library, Web of Science, EBSCOhost, and Embase), where 205 studies were excluded due to duplication. Of the 1743 articles remaining for evaluation, 1679 were excluded because they did not meet the eligibility criteria after reviewing the titles (*n* = 1073) and abstracts (*n* = 606), leaving 64 articles. Subsequently, by reading the full text of the previously selected articles, 51 articles were excluded for not meeting the established inclusion criteria, 24 for including incomplete approaches, 9 for addressing unrelated topics, and 18 for not being RCTs, specifically the reason for the study design, leaving a total of 13 studies [26,27,28,29,30,31,32,33,34,35,36,37,38]. The search and selection process is illustrated in the PRISMA flowchart Figure 1.

### 3.2. Methodological Quality

The methodological quality of the studies included in this systematic review is rated as high, as all 13 studies are RCTs. According to the Oxford Centre for Evidence-Based Medicine (CEBM) classification, they correspond to Level 1a evidence, representing the highest level of methodological rigor for intervention studies.

### 3.3. Risk of Bias

**Figure 2:**
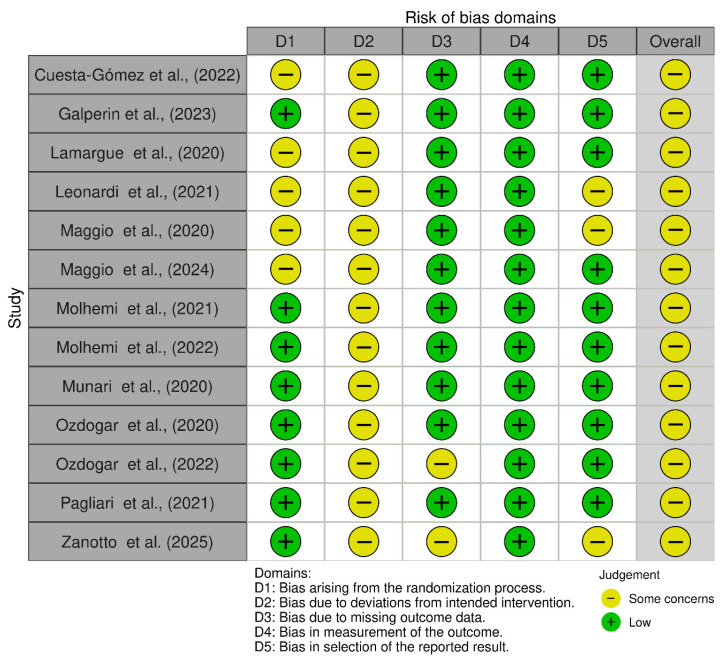
Risk of bias tool: traffic light chart [26,27,28,29,30,31,32,33,34,35,36,37,38].

**Figure 3:**
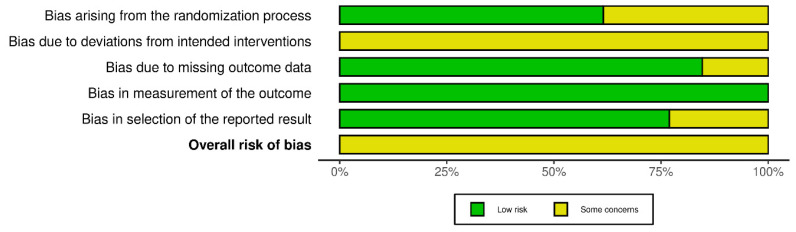
Risk of bias tool: Summary table by domain [26,27,28,29,30,31,32,33,34,35,36,37,38].

Among the included studies, 13 were identified as presenting some concerns regarding methodological quality [26,27,28,29,30,31,32,33,34,35,36,37,38]. These concerns were mainly related to deviations from the intended interventions, incomplete outcome data, and limited reporting of randomization and allocation procedures, which may introduce a moderate risk of bias. Although none of the studies were classified as high risk, the presence of some concerns across all trials indicates methodological uncertainty that affects the confidence in the robustness of the findings. Consequently, the overall certainty of the evidence should be interpreted with caution. All studies were nonetheless retained to preserve the diversity of available evidence and allow a comprehensive evaluation of potential bias effects. Figure 2 and Figure 3 provide a summary of the overall risk-of-bias assessment.

### 3.4. Characteristics of the Studies

Of the 13 included trials, the majority reported favorable effects of VR interventions on function cognitive in people with MS. Significant improvements were observed in information processing speed and memory [27,29,31,35]. The studies can be grouped into three categories: (i) treadmill training with integrated VR [27,38], (ii) exergaming interventions using Kinect or video capture systems [26,30,33,35,36] and (iii) VR combined with conventional or home-based rehabilitation [3,29,31,32,34]. The summary of the characteristics of each study and their main results is given in Table 2.

### 3.5. Sample Characteristics

The pooled sample across all included studies comprised 649 participants diagnosed with MS. The mean age was approximately 40 years, consistent with the demographic most affected by the condition. All trials enrolled individuals with MS, with most participants presenting the chronic stage of the disease. Sample sizes varied notably among studies, ranging from 17 participants in the smallest trial [34] to 124 participants in the largest [31].

### 3.6. Dosages and Interventions Performed

The VR-based interventions exhibited considerable variability in duration, frequency, and intensity among the included randomized controlled trials. Most programs lasted between 6 and 8 weeks, typically delivering 2 to 5 sessions per week of 30 to 60 min each, although some short-term protocols (≤6 weeks) [27,33,36] and longer interventions of up to 17 weeks [28] were also reported. Hospital-based programs integrating VR with conventional occupational or physical therapy were the most frequent [29,32,34], while home-based and telerehabilitation approaches using exergaming platforms such as Kinect or Nintendo Switch were also explored [26,37]. Semi-immersive and non-immersive systems predominated, though treadmill-integrated immersive VR protocols were implemented to target both cognitive and motor functions [27,38]. Additionally, semi-ecological programs such as Urban Daily Cog^®^, described by Lamargue [28], simulated real-life cognitive tasks. Overall, interventions included between 8 and 51 total sessions, reflecting methodological heterogeneity but also highlighting the versatility and adaptability of VR-based rehabilitation across different clinical settings and therapeutic goals.

### 3.7. Cognitive Function

The planned meta-analysis could not be conducted due to the substantial heterogeneity across intervention types, durations, cognitive assessments, and outcome reporting. A narrative synthesis was therefore performed. Several studies reported significant improvements in executive function (PASAT, *p* = 0.012) [34], global cognition (MoCA, *p* < 0.001), and verbal learning and memory (SRT-LTS, *p* < 0.001; WLG, *p* < 0.001) [29]. Improvements in processing speed (SDMT, *p* = 0.001) and verbal learning (CVLT, *p* < 0.05) were also identified [27], with additional gains in processing speed (SDMT, *p* = 0.014), verbal memory (CVLT, *p* < 0.001), and visuospatial memory (BVMT-R, *p* = 0.002) reported by Ozdogar et al. [35] and later confirmed in a follow-up study (*p* < 0.05) [36]. Other trials demonstrated reduced cognitive–motor interference (*p* = 0.038) [32] and improvements in processing speed and executive function (TMT-A/B, *p* < 0.05) [33]. Intragroup improvements in processing speed were also noted (TMT-A, *p* = 0.012), though not accompanied by significant between-group effects [26]. Broader cognitive gains information processing, attention (PASAT-3), verbal memory, visuospatial memory, and cognitive flexibility (TMT-A/B) were reported in additional trials (*p* < 0.05) [28,33]. Further benefits included enhanced cognitive scores within the MSQOL-54 (*p* < 0.001) [30,31] and reduced cognitive frailty (*p* = 0.019) [34].

Overall, the most consistently improved cognitive domains were processing speed, verbal and visuospatial memory, and executive function. Although the evidence supports beneficial effects of VR on cognition in MS, substantial methodological and measurement heterogeneity limits cross-study comparability and adds uncertainty to the overall strength of the findings.

### 3.8. Certainty of Evidence

The certainty of evidence was rated as moderate, supported by consistent findings across several randomized controlled trials (Level 1a, Oxford Centre for Evidence-Based Medicine). However, as no meta-analysis could be performed, this rating represents a narrative estimation of evidence strength rather than a quantitative assessment. Multiple studies reported significant gains in specific cognitive domains, particularly in processing speed, visuospatial memory, and executive function. Nonetheless, overall evidence remains heterogeneous, mainly due to variations in intervention design, sample size, and outcome measures. Therefore, while VR-based interventions demonstrate promising effects on cognitive performance in individuals with MS, the certainty of evidence is still limited, and further high-quality, multicentered RCTs are needed to confirm these preliminary findings (Table 3).

### 3.9. Effects Adverse and Adherence

Across the included RCTs, adherence to VR-based interventions was generally high, with completion rates exceeding 80% in most studies. No serious adverse events were reported, and participants commonly described the sessions as well tolerated. These findings indicate that VR-based rehabilitation is feasible, acceptable, and safe for individuals with MS. Nevertheless, as the overall certainty of evidence was moderate and derived from narrative synthesis rather than meta-analytic data, these results should be interpreted with caution. Future large-scale, multicenter RCTs employing standardized intervention protocols and uniform cognitive outcome measures are required to confirm the safety profile and optimize adherence in clinical settings.

## 4. Discussion

The findings of this review strengthen the growing body of evidence indicating that VR-based interventions can enhance specific cognitive domains in individuals with MS, particularly processing speed, visuospatial memory, and attention. These improvements are likely mediated by the multisensory and feedback-rich nature of VR environments, which promote experience-dependent neuroplasticity and foster engagement through interactive tasks. Integrating cognitive and motor components, as seen in treadmill-based or exergaming interventions, appears especially beneficial, as such approaches simultaneously activate cortical and subcortical networks involved in executive control and dual-task performance. From a clinical perspective, VR-based rehabilitation demonstrates high feasibility, with adherence rates surpassing 80% and an excellent safety profile, supporting its use as an adjunct to conventional cognitive therapy [39]. Its flexibility for home-based application further enhances accessibility for individuals facing mobility or fatigue limitations, aligning with the current shift toward patient-centered, technology-assisted models of care. Nevertheless, the certainty of evidence remains moderate, primarily due to methodological heterogeneity and the lack of meta-analytic data. Future research should aim to develop standardized VR protocols specifying intervention dosage, cognitive targets, and feedback mechanisms, while incorporating neuroimaging and patient-reported outcomes to better elucidate underlying mechanisms and long-term effects. Overall, VR emerges as a safe, feasible, and promising complement to traditional rehabilitation, offering an innovative pathway toward more engaging, individualized cognitive therapy for people with MS.

### 4.1. Cognitive Function

Due to substantial heterogeneity in intervention protocols and cognitive assessment methods, a meta-analysis was not feasible, and consistent effects across trials could not be established. Nevertheless, several individual studies demonstrated measurable benefits. In the single-site RCT evaluating the Memory, Attention, Problem Solving Skills in MS (MAPSS-MS) program, participants exhibited significant improvements in verbal memory and compensatory strategy use compared with controls [40]. These findings were reinforced by a subsequent multisite trial involving 183 participants, which confirmed sustained improvements at six-month follow-up, particularly in processing speed and attention [41]. Similarly, in an RCT including 82 participants, the RehaCom computerized (version 6.10.2, HASOMED GmbH, Magdeburg, Germany) training program produced significant gains in verbal memory on the word list generation task, although effects were not consistent across all cognitive domains [42]. More recent VR-based interventions have also shown benefits in processing speed, visuospatial memory, and dual-task performance [27,29,32,33,35], supporting the growing potential of immersive and technology-assisted approaches to enhance specific aspects of cognition in MS.

Beyond the RCTs included in this review, additional evidence supports the role of VR and cognitively demanding interventions in MS. For instance, treadmill training combined with VR has been shown to improve executive function under dual-task conditions, along with notable motor benefits [38]. Likewise, exergaming approaches have been associated with improvements in processing speed and visuospatial memory, while also increasing motivation and adherence compared with traditional rehabilitation programs [36]. Emerging neurophysiological and molecular evidence further elucidates how VR may facilitate cognitive enhancement through modulation of neural circuitry and synaptic plasticity. Electroencephalographic studies have revealed that VR-based cognitive training elevates alpha-band power in occipital regions and beta-band power in frontal areas, reflecting improved cortical synchronization linked to attention, visuospatial integration, and executive control [43]. At the molecular level, virtual reality experiences promote activity-dependent neuroplasticity by stimulating long-term potentiation and positively regulating brain-derived neurotrophic factor (BDNF), which enhances neuronal connectivity and supports learning-related structural reorganization [44]. Additionally, immersive VR environments have been shown to strengthen functional connectivity within frontoparietal and hippocampal networks, facilitating processes such as working memory, spatial navigation, and cognitive flexibility.

Taken together, these findings suggest that both traditional cognitive rehabilitation programs (e.g., MAPSS-MS, RehaCom) and innovative VR or exergaming interventions can yield meaningful cognitive benefits in individuals with MS. However, evidence remains inconclusive due to variability in sample size, intervention design, and assessment methods. Integrated approaches combining cognitive and motor stimulation appear particularly promising [45,46]. As they promote cortical activation, synaptic efficiency, and ecological validity. Future research should prioritize standardization of protocols and outcome measures to consolidate the evidence base and facilitate the integration of VR into cognitive rehabilitation for people with MS.

### 4.2. Limitations and Strengths

Several limitations should be considered when interpreting the findings of this review. Considerable heterogeneity was observed across intervention protocols in terms of type, duration, frequency, and intensity, limiting comparability between studies. The use of diverse cognitive assessment tools produced inconsistent evidence and hindered standardization across trials. Additionally, many studies included small sample sizes (<40 participants), reducing statistical power and external validity. The absence of standardized intervention parameters such as intensity, frequency, and duration prevented the determination of optimal therapeutic guidelines, and the substantial variability in study protocols, outcome measures, and reporting formats precluded the conduct of a meta-analysis. Finally, some trials exhibited a moderate risk of bias (“some concerns”), underscoring the need for cautious interpretation of the results.

Despite these limitations, this review also presents several strengths. All included studies were randomized controlled trials, providing high-level (1a) evidence. The review followed a rigorous methodology that included comprehensive database searches, predefined eligibility criteria, and independent data extraction by multiple reviewers. Methodological quality and certainty of evidence were systematically assessed using the RoB 2 and GRADE frameworks, ensuring transparency and reproducibility. Moreover, this study provides an updated synthesis of VR-based cognitive interventions MS, addressing an emerging area of rehabilitation science. The included trials demonstrated high feasibility and adherence, with no serious adverse events reported, supporting the safety and acceptability of VR interventions. Finally, by identifying current research gaps, the review offers valuable guidance for future standardized RCTs, and its prospective registration in PROSPERO further strengthens its methodological rigor and credibility.

### 4.3. Practical Applications

The evidence reviewed indicates that VR-based interventions can be safely and feasibly integrated into cognitive rehabilitation for individuals with MS, yielding notable benefits in processing speed and visuospatial memory. In clinical settings, these programs appear most effective when they combine cognitive stimulation with motor engagement, fostering dual-task training and functional transfer. Given the moderate certainty of the evidence, these recommendations should be interpreted as conditional rather than prescriptive, and their application tailored to individual needs, clinical goals, and resource availability. Practically, sessions lasting approximately 30 min and conducted several times per week may include activities such as visuospatial recall, memory-guided step-and-reach exergames, or Stroop-like attention tasks designed to enhance executive function. These formats not only strengthen specific cognitive domains but also emulate the multitasking demands of daily life. Simple VR applications can thus be recommended for self-directed home use, ideally supported by caregivers and reinforced through periodic telemonitoring to maintain continuity of care. Ultimately, VR should be regarded not as a stand-alone modality but as a component of comprehensive, multidisciplinary rehabilitation. Its benefits are maximized when incorporated into coordinated programs led by occupational therapists, physiotherapists, and neuropsychologists, ensuring that cognitive improvements translate into meaningful functional gains and enhanced quality of life.

### 4.4. Clinical Applications

The clinical implementation of VR in MS requires clearly defined assessment and monitoring strategies to ensure that interventions remain effective and adaptable to individual needs. The systematic use of validated cognitive function measures, complemented by physical performance and quality-of-life assessments, allows progress to be tracked at baseline, mid-intervention, and post-intervention, providing essential data for adjusting session frequency, duration, or delivery mode as needed. Given the heterogeneity of cognitive impairment in MS, VR-based rehabilitation should be personalized according to disease stage and functional capacity. For instance, individuals with mild deficits may benefit from more intensive protocols such as exercise-based video games or treadmill-integrated VR whereas those with advanced disability may respond better to shorter, seated programs emphasizing targeted cognitive stimulation. Importantly, VR should be viewed not as a replacement for existing therapies but as a complementary tool within a multidisciplinary framework. In clinical practice, this may involve two supervised VR sessions per week in outpatient settings, supplemented by a home-based session supported by a caregiver. This hybrid approach balances structured professional supervision with home continuity, promoting measurable cognitive improvements, functional transfer, and enhanced quality of life.

## 5. Conclusions

This systematic review indicates that VR-based interventions hold significant potential to enhance cognitive performance in individuals with MS, particularly in domains such as processing speed and visuospatial memory. However, evidence regarding executive function and verbal memory remains inconsistent. The overall certainty of evidence was rated as moderate, limited by heterogeneity in intervention protocols, variability in outcome measures, and small sample sizes across studies. Future research should focus on the development of standardized VR protocols with clearly defined parameters for intensity, frequency, duration, and modality to improve comparability and strengthen the evidence base. Progress in this field will also require consensus-based guidelines and the establishment of core outcome sets tailored to cognitive rehabilitation in MS, ensuring consistency in cognitive, functional, and patient-reported measures across trials. Notably, interventions that integrate cognitive and motor stimulation appear especially promising, as they are associated with higher engagement, strong adherence, and demonstrated feasibility in both clinical and home-based rehabilitation settings.

## Figures and Tables

**Table 1:** Selection criteria used in the systematic review.

Category	Inclusion	Exclusion
Population	Studies involving populations aged 18 years or older, diagnosed with MS.	Studies involving populations whose primary condition is not related to MS (i.e., other neurological disorders).
Intervention	Studies involving cognitive rehabilitation interventions or programs using VR (immersive or non-immersive) for four weeks or more.	Studies using VR for non-rehabilitative purposes (education, assessment only, entertainment, or non-cognitive training).
Comparison	Interventions with an experimental group focused on conventional cognitive rehabilitation.	Absence of control group.
Outcomes	At least one cognitive function assessment.	Studies without baseline data and/or follow-ups.
Study design	RCTs with pre- and post-intervention assessments.	Non-randomized controlled studies, cross-sectional, retrospective, and prospective studies.

MS: Multiple sclerosis; RCTs: Randomized controlled trials; VR: Virtual reality.

**Table 2:** Summary table of results characteristics.

Study	Country	Study Design	Sample	Groups (*n*)	Average Age (Years)	Types of Intervention	Types of VR	Volume Training			Cognitive Function	Main Results
								Weeks	Frequency (Sessions/ Week)	Session Duration (Min)		
[26]	ES	RCT	People with MS	EG: 11 CG: 10	50.9 years old	EG: VR + Conventional CG: Conventional	Non-immersive	8	2	60	TMT-A	↑ TMT-A (*p* = 0.012)
[27]	ISR, US and DE	RCT	People with MS	EG: 62 CG: 62	49.1 years old	EG: Treadmill + VR CG: Treadmill only	Non-immersive	6	3	45	SDMT and CVLT	↑ Cognitive processing speed (SDMT) (*p* = 0.001) ↑ California Verbal Learning Test (CLVT)
[28]	FR	RCT	People with MS	EG: 18 CG: 17	41 years old	SCR vs. NSI	Non-immersive	17	3	45	SDMT, PASAT, CVLT, BVMT-R, TMT-A and TMT-B	↑ Information processing speed (SDMT) (*p* < 0.01) ↑ Attention and processing speed (PASAT-3) (*p* < 0.05) ↑ Verbal memory (CVLT) (*p* < 0.05) ↑ Visuospatial memory (BVMT-R) (*p* < 0.05) ↑ Cognitive flexibility and processing speed (TMT-A and TMT-B) (*p* < 0.05)
[29]	IT	RCT	People with MS	EG: 15 CG: 15	54.6 years old	VR-based cognitive rehabilitation vs. conventional cognitive rehab	Semi-immersive mode	8	3	45	MoCA, SRT-LTS and WLG	↑ Global cognitive functioning (MoCA) (*p* < 0.001) ↑ Learning ability and verbal short-term memory (SRT-LTS) (*p* < 0.001) ↑ Lexical access ability (WLG) (*p* < 0.001)
[30]	IT	RCT	People with MS	EG: 15 CG: 15	39.3 years old	EG: VR + Cognitive training conventional CG: Cognitive training conventional	Semi-immersive mode	8	3	60	MoCA, SRT-LTS, WLG and MSQoL-MT	↑ Global cognitive functioning (MoCA) (*p* < 0.001) ↑ Learning ability and verbal short-term memory (SRT-LTS) (*p* < 0.001) ↑ Lexical-access ability (WLG) (*p* < 0.001) ↑ Quality of life-related to mental state (MSQoL-MT) (*p* < 0.001)
[31]	IT	RCT	People with MS	EG: 35 CG: 35	42.8 years old	VR-based rehabilitation using BTS-Nirvana vs. traditional cognitive rehabilitation	Semi-immersive mode	8	3	60	MSQoL-54	↑ Physical health (*p* < 0.001) ↑ Emotional wellbeing (*p* < 0.001) ↑ Energy (*p* < 0.001) ↑ Health perceptions (*p* < 0.001) ↑ Social functions (*p* < 0.001) ↑ Cognitive function (*p* < 0.001) ↓ Health distress (*p* < 0.001) ↑ Sexual function (*p* < 0.001) ↑ Change in health (*p* < 0.001) ↑ Satisfaction with sexual function (*p* < 0.001) ↑ Overall quality of life (*p* < 0.001) ↑ Physical health—composite score (*p* < 0.001) ↑ Mental health—composite score (*p* < 0.001)
[32]	IR	RCT	People with MS	EG: 19 CG: 20	NR	VR-based balance training using Kinect vs. conventional balance training	Non-immersive	6	3	30	Cognitive-motor performance via dual-task tests	↓ Cognitive-motor interference (Cognitive TUG time) (*p* = 0.038) ↓ Dual-task cost during TUG (DTC-TUG) (*p* = 0.031)
[33]	IR	RCT	People with MS	EG: 18 CG: 18	36 years old	EG: VR CG: Conventional rehabilitation	Non-immersive	6	3	45	TMT-A and TMT-B	↓ TMT-B (*p* < 0.003) and TMT B-A (*p* < 0.002) at post-intervention ↓ SSST at both post-intervention (*p* < 0.002) and follow-up (*p* < 0.04)
[34]	IT	RCT	People with MS	EG: 8 CG: 9	44 years old	Robot-assisted gait training with VR vs. without VR	Non-immersive	6	2	40	PASAT	↑ Executive functions (PASAT) (*p* = 0.012)
[35]	TR	RCT	People with MS	EG: 21 CG: 39	40.1 years old	Video-based exergaming vs. conventional rehab vs. control (no intervention)	Non-immersive	8	1	45	SDMT, CVLT and BVMT-R	↑ Information processing speed (SDMT) (*p* = 0.014) ↑ Verbal memory (CVLT) (*p* < 0.001) ↑ Visuospatial memory (BVMT-R) (*p* = 0.002)
[36]	TR	RCT	People with MS	EG: 15 CG: 15	37.6 years old	Exergaming vs. CR	Non-immersive	8	Diary during hospitalization	45	CVLT and SDMT	↑ Verbal memory (CVLT) (*p* < 0.05) ↑ Information processing speed (SDMT) (*p* < 0.05)
[37]	IT	RCT	People with MS	EG: 35 CG: 35	49 years old	Home-based VR telerehabilitation vs. conventional home-based rehab	Semi-immersive mode	6	5	45	MoCA, SRT-LTS, SRT-CLTR, SRT-DR, SPART and PASAT	↑ Global cognitive functioning (MoCA) (*p* = 0.046) ↑ Verbal memory (SRT-LTS) (*p* = 0.002) ↑ Verbal memory (SRT-CLTR) (*p* = 0.030) ↑ Verbal memory (SRT-DR) (*p* = 0.006) ↑ Visuospatial memory (SPART) (*p* = 0.007) ↑ Attention/processing speed (PASAT) (*p* = 0.004)
[38]	US, ISR and DE	RCT	People with MS	EG: 44 CG: 39	49 years old	Treadmill training with VR vs. treadmill training only	Non-immersive	6	3	30–40	FI-Cognitive (subset of frailty index)	Treadmill + VR Group (TT + VR): ↓ Cognitive frailty index (FI-Cognitive) (*p* = 0.019) ↓ Overall frailty index (FI-total) (*p* = 0.002) frailty in TT + VR compared to TT (*p* = 0.019)

BVMT-R: Brief Visuospatial Memory Test–Revised; BTS-Nirvana: Biomechanical Training System—Nirvana; CG: Control Group; CR: Conventional Rehabilitation; CVLT: California Verbal Learning Test; DE: Germany; ES: Spain; FR: France; FI-total: Overall Frailty Index; IR: Iran; ISR: Israel; IT: Italy; TR: Turkey; US: United States; EG: Experimental Group; FI-Cognitive: Frailty Index—Cognitive Subset; MoCA: Montreal Cognitive Assessment; MS: Multiple Sclerosis; MSQoL-MT: Multiple Sclerosis Quality of Life—Mental Health domain; MSQoL-54: Multiple Sclerosis Quality of Life-54; NSI: Non-Specific Intervention; PASAT: Paced Auditory Serial Addition Test; RCT: Randomized Controlled Trial; SCR: Specific Cognitive Rehabilitation; SDMT: Symbol Digit Modalities Test; SPART: Spatial Recall Test; SRT-CLTR: Selective Reminding Test—Consistent Long-Term Retrieval; SRT-DR: Selective Reminding Test—Delayed Recall; SRT-LTS: Selective Reminding Test—Long-Term Storage; SSST: sixspot step test; TMT-A: Trail Making Test Part A; TMT-B: Trail Making Test Part B; TMT B–A: Difference score between Trail Making Test Part B and Part A; TT: Treadmill Training; TUG: Timed Up and Go; DTC-TUG: Dual-Task Cost during Timed Up and Go; VR: Virtual Reality; WLG: Word List Generation; ↑: indicates a statistically significant improvement/increase in the reported outcome; ↓: indicates a statistically significant reduction/decrease in the reported outcome.

**Table 3:** Evaluation of methodological quality using the GRADEpro tool.

Certainty of Evidence							No of Patients		Effect		Certainty
No of Studies	Study Design	Risk of Bias	Inconsistency	Indirect Evidence	Vagueness	Other Considerations	[Intervention]	[Comparison]	Relative (95% CI)	Absolute (95% CI)	
13	RCT	Serious	Not serious	Not serious	Not serious	None	337/649 (51.9%)	312/649 (48.1%)	Not estimable		+++ Moderate

CI: Confidence interval; RCT: Randomized clinical trial; +++: Moderate Certainty.

## Data Availability

All data generated or analyzed during this study are included in this published article.

## Supplementary Materials

The following supporting information can be downloaded at: https://www.mdpi.com/article/10.3390/jcm15124534/s1, Table S1: PRISMA 2020 Checklist.
